# Effects of Rhythm and Rate-Controlling Drugs in Patients With Permanent His-Bundle Pacing

**DOI:** 10.3389/fcvm.2020.585165

**Published:** 2020-12-17

**Authors:** Lan Su, Xue Xia, Dongjie Liang, Shengjie Wu, Lei Xu, Tiancheng Xu, Songjie Wang, Xiao Chen, Weijian Huang

**Affiliations:** ^1^Department of Cardiology, the First Affiliated Hospital of Wenzhou Medical University, Wenzhou, China; ^2^The Key Lab of Cardiovascular Disease, Science and Technology of Wenzhou, Wenzhou, China

**Keywords:** his bundle pacing (HBP), antiarrhythmic drugs, H-V conduction, safety, pacing parameters, rhythm and rate-controlling

## Abstract

**Introduction:** Antiarrhythmic drug therapy can affect pacemaker parameters in both the atrial and ventricular myocardium. It is not known whether antiarrhythmic drugs impact His bundle pacing/sensing parameters and His to ventricle (H-V) intervals following permanent His bundle pacing (HBP). The aims of the study were to prospectively determine the influence of rhythm and rate-controlling drugs on pacing parameters and H-V conduction after His bundle lead implantation and to assess the impact of rhythm and rate-controlling drugs on the safety of HBP.

**Materials and Methods:** Patients (*N* = 140) with QRS duration < 120 ms who met permanent pacing indications were prospectively enrolled. Propafenone, lidocaine, and adenosine were injected intravenously after implantation of 3,830 lead during the procedure. Metoprolol succinate, amiodarone, and digoxin were taken orally for 1 month. Pacing parameters before and after drug intervention was measured, including His capture threshold, sensing and impedance, H-V interval, and conduction.

**Results:** There were no statistically significant differences in His bundle pacing thresholds, impedance, and sensing after drug intervention at implantation or during a 2-month follow-up (*P* > 0.05). The HV interval was not affected except in the large-dose propafenone group where HV interval prolonged (*P* = 0.001). All patients maintained 1:1 H-V conduction following drug administration.

**Conclusion:** There was no adverse impact on the HBP parameters or H-V conduction after the administration of commonly used dosage of rhythm and rate-controlling drugs. The drugs were safe in patients with permanent His bundle pacing.

## Introduction

Arrhythmias are common in patients with pacemakers and may require management with antiarrhythmic drugs (AADs), which may affect the cardiac conduction system (1–3). Previous studies (4–7) have reported that some AADs affect atrial and ventricular pacing threshold and impedance. Others have reported that AADs affect defibrillation thresholds of implantable cardioverter defibrillators (8, 9).

Recently, the feasibility and effectiveness of permanent His-bundle pacing (HBP) has been demonstrated in patients with indications for ventricular pacing or cardiac resynchronization therapy (CRT) (10–13). During the last decade, permanent HBP has increasingly been adopted in clinical practice with the availability of Select Secure™ lead (model 3830, Medtronic, Inc. Minnesota, USA) and the delivery sheath (model C315His and/or C304, Medtronic, Inc., Minnesota, USA) (14–16). The tip of 3,830 lead can be implanted in the conduction system to selectively pace the His bundle.

The aim of our study was to determine the effects of rhythm and rate-controlling drugs on acute and chronic His capture thresholds, impedance, and sensing of HBP lead, as well as infra-Hisian conduction during and after permanent HBP lead implantation. This is the first study to test the changes of pacing parameters and conductivity after HBP.

## Materials and Methods

### Study Design

This was a prospective, single-center study from January 2016 to September 2020. Consecutive patients who had indications for permanent pacemaker implantation and underwent successful permanent HBP were enrolled. Informed written consent was obtained prior to enrollment. The institutional review board approved the study protocol. Exclusion criteria were as following: (1) patients with infra-Hisian AV block or QRS duration ≥120 ms, except right bundle block pattern; (2) patients who took AADs up to 7 days before implantation; (3) patients with a history of allergy or intolerance to AADs; (4) women of child-bearing age with no reliable contraception; and (5) fluctuation in acute His bundle capture thresholds during the implantation procedure with a change in threshold of more than 0.3 V/0.5 ms; fluctuation of immediate and 1-month threshold for His bundle capture of more than 0.5 V/0.5 ms.

The criteria of successful HBP: (1) His capture threshold during procedure is 2.0 V/0.5 ms; and (2) R-wave amplitude is >2.5 mv without far-field atrial oversensing.

### Materials

The Select-Secure 3830^TM^ (Medtronic, Minneapolis, MN) lead and its specific delivery sheath C315 or C304 were used in all patients. Intra-procedural parameters were measured using the pacing system analyzer (Model 2290, Medtronic, Inc., Minnesota, USA). A 12-lead surface electrocardiogram (ECG) and intracardiac electrograms (EGMs) were recorded with multichannel electrophysiological monitor (GE Cardio Lab EP Recording System 2000, General Electric Inc., Wisconsin, USA). A post-procedural 12-lead ECG was recorded when subjects underwent pacemaker programming. The His bundle capture threshold and pacing impedance measurements were performed at a pulse width of 0.5 ms in VVI mode.

### Subgroup of Patients and Route of Administration of Rhythm and Rate-Controlling Drugs

The patients with successful HBP were subdivided into intravenous drug intervention group and oral drug intervention group.

#### Intravenous Drug Intervention Group (Intravenous Group, *n* = 70)

If the His bundle capture threshold fluctuation was <0.3 V/0.5 ms at 10 min after lead fixation, it was considered stable. All patients had no contraindication to drugs tested. One of the following drugs was injected: (1) propafenone 70 mg (*n* = 10) or 140 mg (*n* = 10) intravenously over 5 min; (2) 0.75 mg/Kg or 1.5 mg/Kg lidocaine (*n* = 20, *n* = 10) intravenously over 5 min; or (3) 9 mg adenosine (*n* = 20) as an intravenous bolus. Pacing thresholds, impedance, and sensing were measured in unipolar and bipolar configurations at the same time of post-injection simultaneously and quickly. The His to ventricular conduction(HVC)was measured from the pacing lead by pacing at high rates (>140 bpm) and at His capture voltage output range. The 12-lead surface ECG and intracardiac electrograms were recorded simultaneously during the testing.

#### Oral Drug Intervention Group (Oral Drug Group, *n* = 70)

At the 1-month follow-up, pacing parameters including threshold, sensing, and impedance in unipolar and bipolar configurations along with HVC (HV interval) were measured to evaluate the lead stability. The stability criterion was set as the difference between His bundle capture threshold at 1 month and implant to be <0.5 V/0.5 ms. One of three oral drugs amiodarone (*n* = 20), β-blocker(*n* = 40), or digoxin (*n* = 10) was administered for 1 month. Selection of drug and dosage was based on clinical requirements such that patients using beta-blockers and amiodarone had atrial arrhythmias, such as paroxysmal atrial tachycardia, or atrial fibrillation, while digoxin was used in patients with ejection fraction (EF) <50%. All parameters were rechecked after 1 month of continuous oral drug therapy.

### Measurement of Pacing Parameters

#### Threshold

His bundle capture threshold was tested by unipolar at 0.5 ms pulse width and 12 lead surface ECG and intracardiac electrograms were recorded simultaneously during all measurements.

#### Sensing

R-wave amplitude in the HBP lead was measured in the setting of bipolar configuration. Amplitudes of the R wave (when present) was performed manually with real-time display from the programmer screen and printed. Measurements were repeated three times in each configuration to obtain mean data.

#### Impedance

Average value of impedances tested three times at HBP tip-ring configuration was defined as the final impedance value.

#### H-V Conductivity

The H-V interval was measured and HVC was tested at the range of threshold output voltage no more than 0.3 V/0.5 ms above the HBP threshold. The HV conduction was defined as normal if the 1:1 conduction was demonstrable at pacing rates of more than 140 beats per minute (bpm).

### Data Collection and Follow-Up

Baseline demographic data, medical history, and medications were obtained at the time of enrollment. Outpatient follow-ups were conducted at 1 and 2 months, respectively, after the successful implantation of the His bundle lead and the pacing parameters, including His capture thresholds, sensing, and impedance were collected.

### Data Analysis

Continuous data were expressed as mean ± SD. The unpaired or paired *t*-test was used to compare the differences of mean values between two groups or two time-points in the same group if the data were normally distributed. Categorical data were described as number (%), and χ^2^ test or Fisher's exact test was used to determine the differences between groups. All analyses were performed using the SPSS version 22.0 (IBM Corp., Armonk, NY, USA). All analyses were two-sided and *P*-value < 0.05 was considered significant.

## Results

During the study period, HBP was attempted in 167 patients with bradycardia pacemaker indications and successful in 156 patients (93.4%) according to the HBP criteria. Based on the stable criteria for HBP lead, there were 140 patients (89.7%) included in and divided into intravenous or oral drug groups.

**Figure 1:**
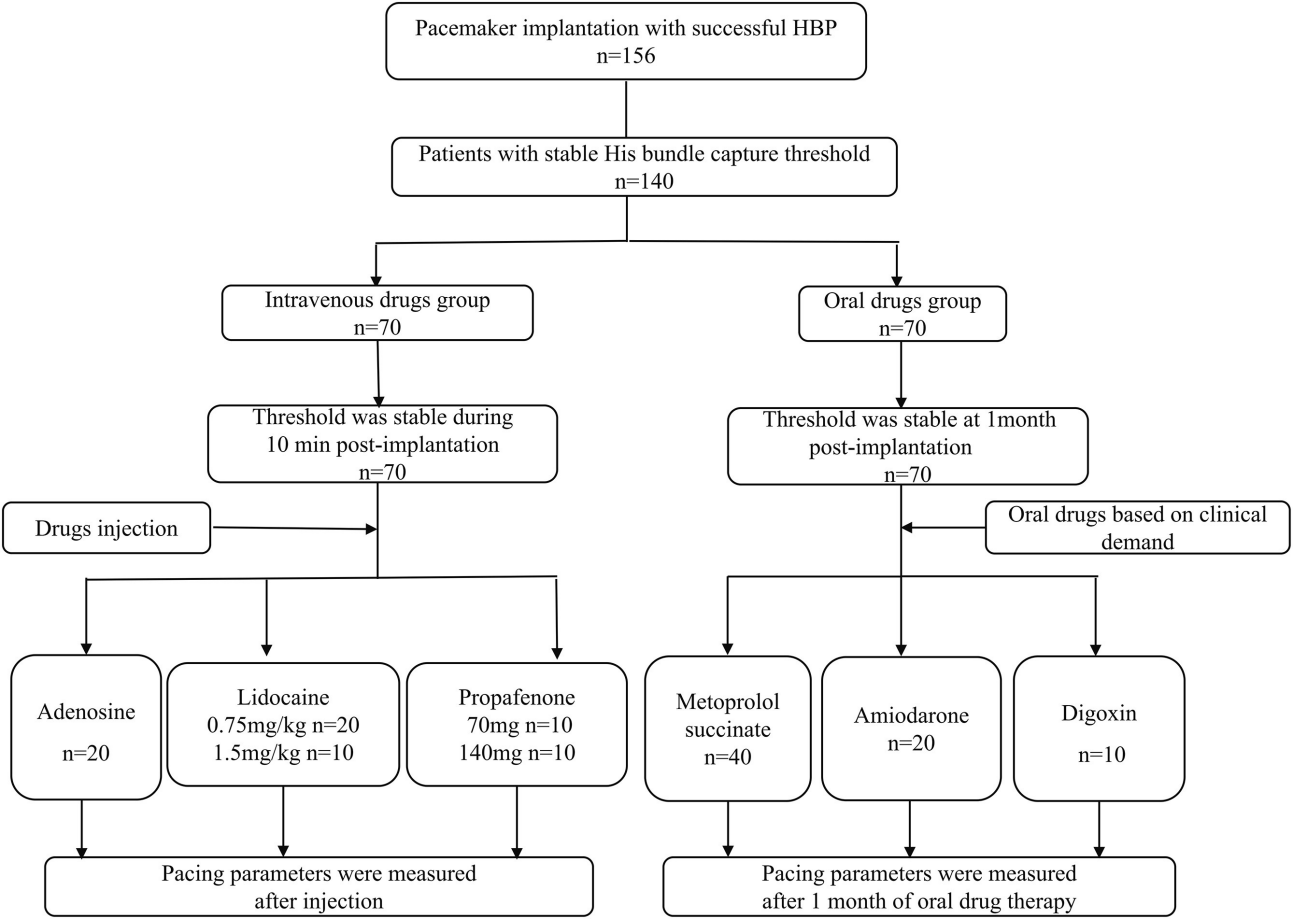
Flow chart of patients and route of administration of rhythm and rate-controlling drugs.

A flow chart of patients and route of administration of drugs are displayed in Figure 1. Baseline characteristics of the subjects are shown in Table 1. There were 36 patients (25.7%) with sick sinus syndrome and 104 with atrioventricular block (74.3%) enrolled in the study. The intrinsic QRS duration was 104.6 ± 32.8 ms.

**Table 1:** Baseline characteristics of the subjects.

**Parameters**	**Intravenous group (*n* = 70)**	**Oral group (*n* = 70)**
Male	34 (48.5%)	43 (61.4%)
Age	72.4 ± 10.7	66.5 ± 9.9
Weight (kg)	63.5 ± 13.0	63.5 ± 9.4
Hypertension	26 (37.1%)	35 (50.0%)
DCM	3 (4.3%)	12 (17.1%)
HCM	1 (1.4%)	3 (4.3%)
ICM	0 (0.0%)	1 (1.4%)
CAD	4 (5.7%)	14 (20.0%)
AF	33 (47.1%)	44 (62.8%)
Paroxysmal	9 (12.8%)	14 (20.0%)
Persistent	24 (34.3%)	30 (42.8%)
**INDICATION OF PACING**		
SSS	14 (20.0%)	22 (31.4%)
AVB	56 (80.0%)	48 (68.6%)
Intrinsic QRS duration, ms	100.2 ± 31.8	107.8 ± 34.7
HV interval, ms	50.4 ± 8.2	52.8 ± 10.3
**ECHOCARDIOGRAM**		
LVEF, %	57.1 ± 15.7	52.6 ± 17.5
LVEDd, mm	54.1 ± 10.3	56.9 ± 11.3
LAD, mm	47.3 ± 7.2	49.3± 9.7
**MEDICATIONS**		
Anticoagulant	34 (48.5%)	30 (42.8%)
Antiplatelet	12 (17.1%)	30 (42.8%)
ACEI/ARB	30 (42.8%)	53 (75.7%)
Diuretic	21 (30.0%)	35 (50.0%)
Statins	32 (45.7%)	39 (55.7%)

*DCM, dilated cardiomyopathy; ICM, ischemic cardiomyopathy; HCM, hypertrophic cardiomyopathy; CAD, coronary artery disease; SSS, sick sinus syndrome; AF, atrial fibrillation; AVB, atrioventricular block; AF, atrial fibrillation; AVB, atrioventricular block*.

As shown in Table 2, three drugs were administrated during the procedure including 9 mg adenosine, of 70 and 140 mg propafenone, and 0.75 and 1.5 mg/kg lidocaine in the intravenous group. There were no statistical differences of HBP thresholds, sensing, and impedances following drug administration in unipolar or bipolar configurations. The results showed that the above three drugs had no effects on the measured parameters (*P* > 0.05). There was statistical difference in native HV interval (47.6 ± 5.4 vs. 53.6 ± 6.1 ms, *P* = 0.001) in the 140 mg propafenone group, but only a mild prolongation of HV interval without statistical difference (49.7 ± 6.7 vs. 53.1 ± 4.9, *P* = 0.061) in the 70 mg propafenone group. The immediate HV conduction remained 1:1 during pacing at 140 bpm at threshold range output after intravenous injection in all patients.

**Table 2:** Effects of drugs on pacing parameters in intravenous group and oral group.

**Drugs**	**Sensing**, **mV**		**Threshold**, **V/0.5ms**		**Impedance**, **Ω**		**HV interval, ms**
	**Unipolar**	**Bipolar**	**Unipolar**	**Bipolar**	**Unipolar**	**Bipolar**	
**INTRAVENOUS GROUP (AT IMPLANTATION**, ***n*** **=** **70)**							
**Lidocaine 0.75 mg/kg (*****n*** **=** **20)**							
Before	4.36 ± 1.96	3.73 ± 2.25	1.25± 0.88	1.47 ± 0.98	422.8 ± 73.6	521.5 ± 88.0	49.4 ± 8.7
After	4.19 ± 1.66	3.38 ± 2.02	1.27± 0.78	1.51 ± 1.02	422.2 ± 96.8	504.1 ± 76.3	49.7 ± 7.8
*P*	0.336	0.154	0.717	0.455	0.962	0.124	0.437
**Lidocaine 1.5 mg/kg (*****n*** **=** **10)**							
Before	3.93 ± 2.28	3.88 ± 2.61	1.36 ± 0.57	1.63 ± 0.56	557.5 ± 125.0	630.0 ± 106.9	50.0 ± 7.6
After	4.03 ± 2.68	3.86 ± 2.75	1.29 ± 0.51	1.53 ± 0.50	552.3 ± 114.6	625.0 ± 96.2	49.5 ± 7.7
*P*	0.729	0.955	0.738	0.615	0.653	0.582	0.582
**Adenosine 9 mg (*****n*** **=** **20)**							
Before	3.78 ± 1.71	3.59 ± 1.61	1.04 ± 0.90	1.31 ± 1.06	438.0 ± 68.9	515.4 ± 69.1	54.9 ± 9.8
After	3.87 ± 1.59	3.54± 1.68	1.10 ± 0.88	1.36 ± 1.07	427.8 ± 68.6	516.7 ± 70.4	54.5 ± 8.7
*P*	0.461	0.456	0.219	0.164	0.227	0.725	0.420
**Propafenone 70 mg (*****n*** **=** **10)**							
Before	8.25 ± 6.32	6.88 ± 6.56	0.51 ± 0.15	0.73 ± 0.22	550.9 ± 142.3	653.7 ± 148.8	49.7 ± 6.7
After	6.17 ± 3.26	7.85 ± 6.76	0.51 ± 0.16	0.60 ± 0.14	547.0 ± 145.0	614.6 ± 117.1	53.1 ± 4.9
*P*	0.280	0.748	1	0.243	0.914	0.234	0.061
**Propafenone 140 mg (*****n*** **=** **10)**							
Before	4.24 ± 1.82	4.08 ± 2.46	1.01 ± 0.50	1.32 ± 0.52	506.3 ± 107.6	588.8 ± 102.3	47.6 ± 5.4
After	4.50 ± 2.27	5.03 ± 3.41	0.91 ± 0.36	1.21 ± 0.38	491.6 ± 101.0	588.8 ± 103.6	53.6 ± 6.1
*P*	0.432	0.120	0.200	0.158	0.140	1.000	0.001
**ORAL GROUP (1 MONTH FOR DRUGS**, ***n*** **=** **70)**							
**Metoprolol succinate (*****n*** **=** **40)**							
Before		5.76 ± 5.09		1.09 ± 0.72		463.3 ± 61.9	
After		6.04 ± 5.67		1.13 ± 0.82		458.4 ± 58.0	
*P*		0.771		0.456		0.631	
**Amiodarone hydrochloride (*****n*** **=** **20)**							
Before		3.45 ± 3.05		1.31 ± 0.66		520.0 ± 147.0	
After		4.02 ± 4		1.43 ± 0.77		460.0 ± 55.2	
*P*		0.198		0.457		0.209	
**Digoxin (*****n*** **=** **10)**							
Before		3.18 ± 2.06		1.14 ± 0.86		467.1 ± 61.2	
After		2.73 ± 2.83		1.16 ± 0.58		448 ± 41.7	
*P*		0.401		0.956		0.490	

If HBP thresholds remained stable at 1-month post-implant, one of the following three oral drugs was given to the patients in the oral group: metoprolol succinate (47.5/95 mg qd), amiodarone hydrochloride (after loading dose 600 mg per day for 7 days, maintenance dose 200 mg qd) and digoxin (0.125 mg qd). Results showed no statistical differences in pacing thresholds, impedance and sensing after either drug was administered in patients whose threshold remained stable at 1-month post-implant (Table 2). Compared with the QT intervals before and after 1-month drug intervention in amiodarone group, statistical difference was observed (0.433 ± 0.044 vs.0.454 ± 0.050 s, *P* = 0.03). The HBP percentage with three oral drugs increased from 63.7 ± 42.2% to 76.0 ± 38.3% (*P* = 0.003) after drug intervention.

## Discussion

Efficacy and long-term safety of pacing are very important in pacemaker-dependent patients. Since the clinical application of HBP in 2000 (10), many studies have demonstrated that the pacing parameters of HBP are different from conventional ventricular pacing, showing lower sensing, higher acute pacing thresholds, and significant rise in long-term pacing thresholds (15). However, some investigations have reported the pacing thresholds of HBP could be lower than 1 V (16–19); while others have reported fluctuations in His capture threshold. A meta-analysis revealed that the average pacing threshold at implant of HBP was 1.73 V and 1.79 V at >3 months follow-up with 3,830 active fixation leads (20).

Patients with permanent pacemakers often need AADs or medications for heart failure treatment in clinical practice. Previous literature has shown that antiarrhythmic agents such as class IA and class III drugs might affect pacing parameters and increase pacing thresholds in conventional pacing sites. To our knowledge, the effects of a full range of AADs on permanent HBP capture thresholds have not been previously systematically studied. The His bundle area can be located precisely using the 3,830 lead, and physiological pacing can be achieved by capturing the conduction system. Due to the special electrical characteristics of the His Purkinje system, we investigated the effect of rhythm and rate-controlling drugs, which might influence the pacing parameters and especially HVC. The results of our study indicate that the drugs we tested had no significant effects on the acute and chronic pacing parameters of the His bundle lead.

**Figure 2:**
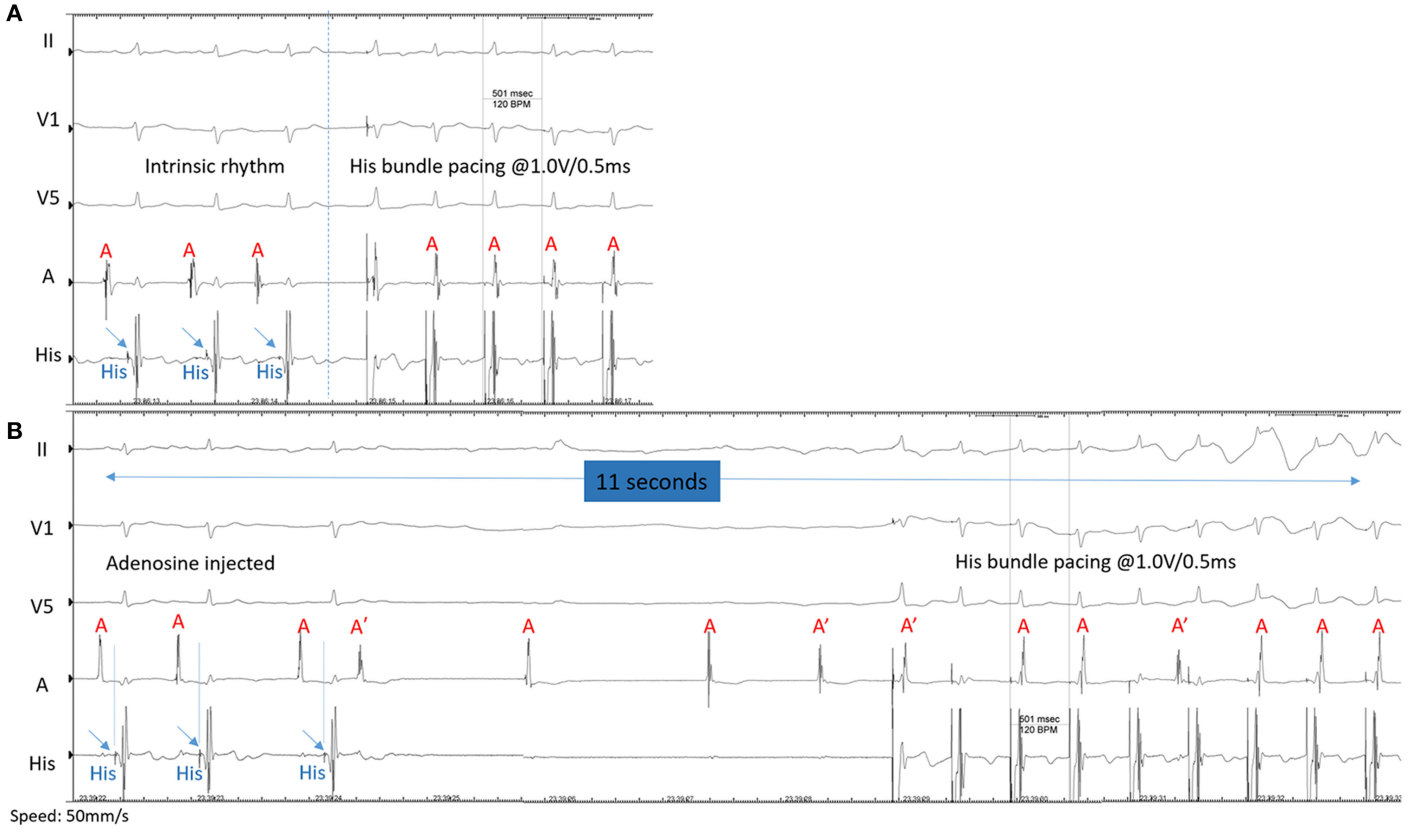
**(A)**The intrinsic surface ECG and EGM show sinus rhythm with His-ventricle (H-V) interval of 45 ms. Pacing is performed at 1.0 V @ 0.5 ms with 120 bpm to ensure selective His bundle capture. The last four beats demonstrated 1:1 retrograde conduction to the atrium. **(B)** After intravenously injection of 9 mg of adenosine, the conduction from atrium to His bundle prolonged gradually as Wenckebach conduction occurred and eventually caused complete AV nodal block, above the His bundle. Simultaneous pacing at 1.0 V @ 0.5 ms with 140 bpm captures the His bundle with 1:1 antegrade H-V conduction. The last three beats showed recovery of 1:1 H-A retrograde conduction.

Similar to a previous study (21), there was significant prolongation of the HV interval in the large dose propafenone group in our study. We also found that the drugs used in all patients had no effect on HV conduction below pacing site. These findings also confirmed that the pacing lead implanted in the His bundle was beyond the AV junction. Figure 2 shows that the conduction above the His bundle can be blocked by adenosine with resultant transient AV nodal block, while the conduction below the His bundle lead was not affected and still maintained 1:1 HVC at more than 140 bpm pacing. Our study provided the experimental evidence for clinical safety and usage of these drugs in patients with permanent HBP.

HBP achieved optimal cardiac synchronization but is limited by suboptimal lead delivery and high thresholds. Recently, another form of conduction system pacing, left bundle branch pacing (LBBP), has been demonstrated to have better and stable pacing parameters than that of HBP (22–24). It is possible that AADs may not significantly affect the pacing parameters in LBBP due to pacing beyond the site of block. However, the stability of pacing parameters following AADs in patients with LBBP should be assessed in future studies.

## Limitations

This was a single center, non-randomized study. Small sample size and failure to analyze other AADs are additional limitations of the study. The sample size of patients who took metoprolol succinate can achieve more than 90% statistical power at a two-sided 0.05 significance level to detect the threshold difference of 0.5 V or larger, which is considered as a clinically significant difference. The actual threshold difference is <0.5 V, considered as no clinically significant difference. However, the negative conclusions may not be drawn in all groups due to the small sample size.

## Conclusion

In summary, we did not demonstrate any significant changes in His bundle capture thresholds, impedance, and R wave amplitudes, after acute intravenous or after 1-month oral administration of commonly used rhythm and rate-controlling drugs. On the other hand, the electrophysiological conduction below the His bundle lead was not affected at the dosage studied. We conclude that the routine dose of AADs studied is safe in patients with permanent HBP, especially in those who are pacemaker dependent.

## Data Availability Statement

The original contributions presented in the study are included in the article/supplementary material, further inquiries can be directed to the corresponding author/s. Requests to access the datasets should be directed to weijianhuang69@126.com.

## Ethics Statement

The studies involving human participants were reviewed and approved by Clinical Research Ethics Committee of the First Affiliated Hospital of Wenzhou Medical University. The patients/participants provided their written informed consent to participate in this study.

## Author Contributions

LS: design, definition of intellectual content, clinical studies, manuscript preparation, manuscript editing. XX: manuscript preparation, manuscript editing. DL, ShW, and LX: data acquisition, data analysis, statistical analysis. TX, SoW, and XC: literature search. WH: design, definition of intellectual content, clinical studies, manuscript preparation, manuscript editing, manuscript review. All authors contributed to the article and approved the submitted version.

## Conflict of Interest

The authors declare that the research was conducted in the absence of any commercial or financial relationships that could be construed as a potential conflict of interest.
